# Validation of a single factor representing the indicators of metabolic syndrome as a continuous measure of metabolic load and its association with health and cognitive function

**DOI:** 10.1371/journal.pone.0208231

**Published:** 2018-12-12

**Authors:** Sandra Düzel, Nikolaus Buchmann, Johanna Drewelies, Denis Gerstorf, Ulman Lindenberger, Elisabeth Steinhagen-Thiessen, Kristina Norman, Ilja Demuth

**Affiliations:** 1 Center for Lifespan Psychology, Max Planck Institute for Human Development, Berlin, Germany; 2 Charité – Universitätsmedizin Berlin, corporate member of Freie Universität Berlin, Humboldt-Universität zu Berlin, and Berlin Institute of Health, Lipid Clinic at the Interdisciplinary Metabolism Center, Berlin, Germany; 3 Humboldt University Berlin, Berlin, Germany; 4 Charité – Universitätsmedizin Berlin, corporate member of Freie Universität Berlin, Humboldt-Universität zu Berlin, and Berlin Institute of Health, Research Group on Geriatrics, Berlin, Germany; 5 German Institute of Nutrition Potsdam-Rehbrücke, Dept. of Nutrition and Gerontology, Nuthetal, Germany; 6 Berlin-Brandenburg Center for Regenerative Medicine (BCRT), Charité Universitätsmedizin Berlin, Germany; West Virginia University, UNITED STATES

## Abstract

The metabolic syndrome (MetS) is a cluster of risk factors for cardiovascular disease associated with reduced physical fitness, higher disease burden, and impaired cognitive functions. Little is known about the operation of these risk factors in older adults when considered comprehensively without relying on the cut-off values of the single MetS components. The three main aims of the current study were to: (i) establish a latent metabolic load factor (MetL), using confirmatory factor analysis (CFA), and representing a continuous measure of MetL, defined by indicators that are commonly used to separate MetS groups from healthy individuals; (ii) examine the associations of this MetL factor with objective health, and cognitive function in men and women; (iii) compare the magnitude of these associations to those observed for the individual indicators used to define the MetL factor as well to the classical categorized MetS vs. non-MetS groups. The current analysis is based on cross-sectional data from 1,609 participants of the Berlin Aging Study II (mean age = 68.5 years, SD (3.7); 50.1% female). We applied structural equation modeling (SEM) to establish a latent MetL factor defined by the five indicators commonly used to diagnose MetS. The latent MetL factor was associated with physician-assessed morbidity and kidney function (estimated glomerular filtration rate, eGFR) in both men and women, but not with hand grip strength and lung function (Forced Expiratory Volume in 1 Second (FEV1)). In addition, we found a negative association between MetL and fluid intelligence among men. A continuous latent variable approach representing the common variance of MetS indicators is well suited to foster our understanding of human aging as a systemic phenomenon in which risk factors are operating on either side of the normal versus pathological divide.

## Introduction

The metabolic syndrome (MetS) defines a cluster of medical conditions (elevated triglycerides, low high-density-lipoprotein-cholesterol (HDL-C), abdominal obesity, insulin resistance and elevated blood pressure), which is associated with increased risk of cardiovascular disease and has a prevalence increasing with age [1,2]. Moreover, several associations between MetS and non-cardiovascular phenotypes such as osteoporosis, vitamin D deficiency, chronic kidney disease, reduced physical fitness as indicated by lower grip strength and lung function (Forced Expiratory Volume in 1 Second, FEV1) have been observed [3–8]. Notably, FEV1 and hand grip strength have been shown to be excellent predictors of short and long-term outcomes such as mortality, disability or need for care [9–13]. In addition, some studies investigating the relationship between MetS and cognitive capacity in patient- or population-based cohorts have found that cognitive performance is impaired in individuals diagnosed with MetS, particularly in relation to executive functions [14,15]. Altogether, the majority of studies point to a negative association of MetS with cognitive performance and brain structure, which is commonly attributed to impaired vascular reactivity, neuroinflammation, oxidative stress or abnormal brain lipid metabolism [14].

The comparability between studies reporting MetS associations with health indicators and cognitive performance is, however, currently restricted due to the variety of MetS definitions with variable cut-off values resulting in heterogeneous study groups. A common statement for the definition of MetS was published in 2009 by Alberti et al., suggesting that any three out of the five MetS-criteria have to be fulfilled to be diagnosed with MetS [1]. Though there is a broad consensus on this definition, the mechanisms of MetS are still controversially discussed. There is a debate on how single indicators relate to the syndrome and which pathogenesis is central to MetS. Also, fixed cutoff values are difficult to apply to all individuals. As a result, severity of condition such as intensity and consistency of metabolic impairment can vary between subjects given the same MetS diagnosis, obscuring the heterogeneity of the condition [16]. Although differences in men and women with respect to the MetS components and the development of arteriosclerosis have been recognized, cutoffs for MetS scores are generally not sex-specific, apart from HDL-C and waist circumference [17–19]. In recent years, studies have tried to address the methodological limitations evoked by categorical cutoffs by including different approaches such as exploratory and confirmatory factor analyses (EFA; CFA, respectively) to include full continuous information of single indicators to define composites of MetS. CFA is a statistical approach to verify a sound theoretical concept that is based on empirical data or theoretical pathways and has already been used in the context of the MetS [20,21].

Thus, the main purpose of the present study was to model a latent factor employing CFA representing *metabolic load* by considering the five indicators as continuous variables that represent the concept of the MetS [1] and making use of cross-sectional data from generally physically and cognitive healthy older participants of the Berlin Aging Study II (BASE-II). This allowed us to investigate in a next step the predictive validity of a continuous *metabolic load* factor with respect to physical health indicators and cognitive function. Finally, we investigated the group differences within the categorial MetS definition with respect to physical health and cognitive function.

## Methods

### Participants and study design

The study population investigated here consisted of 1,609 healthy older participants of the BASE-II study (mean age = 68.5 years, SD (3.7); 50.1% female) with valid data on MetS diagnosis. Participants were recruited from the greater metropolitan area of Berlin via advertisements in regional newspapers and public transportation systems as well as through a participant pool at the Max Planck Institute for Human Development. The medical examination consisted of a two-day protocol including a comprehensive anamnesis performed by a physician and a wide array of laboratory and functional tests. Individuals participated in two cognitive testing sessions scheduled one week apart, and were tested in small groups (e.g., about six participants per group [22,23]). BASE-II was approved by the ethics committee of the Charité-Universitätsmedizin Berlin (number of the ethical approval: EA2/029/09), and by the ethics committee of the Max-Planck-Institute for Human Research. All of the participants provided written informed consent. A detailed description of the overall study design, sampling methods, variables, and procedures has been described earlier in detail [22,23].

### Selection of MetS variables to define a metabolic load factor

We selected only the indicators based on the joint statement on MetS of the International Diabetes Federation Task Force [1]: Waist circumference (waist), High Density Lipoprotein (HDL), fasting blood glucose (BG1), triglycerides (TG), and systolic/diastolic blood pressure. Waist circumference was assessed using a tape measure at the level of the umbilicus. Systolic/diastolic blood pressure was measured in a sitting position on the left arm as part of a medical examination, using an electronic sphygmomanometer (boso-medicus memory, Jung Willingen, Germany). After a fasting period of at least 8 hours, blood was collected from the subjects, subsequently stored at 4–8°C and prepared for transport and subsequent measurement on the same day. The laboratory parameters were analyzed by a certified laboratory (Labor 28 GmbH, Berlin and Labor Berlin). Serum triglycerides and high-density lipoprotein (HDL) cholesterol were measured with enzymatic color tests (Roche/Hitachi Modular; device: ACN 435 und ACN 781). Glucose levels (fasting) were measured using photometric methods and an oral glucose tolerance test (OGTT) was performed according to the WHO-guidelines in subjects without self-reported diabetes mellitus type II (T2D) [23,24].

### Objective health variables

Physical fitness represents one’s state of general muscle strength, musculoskeletal capacity, and general vitality and has been repeatedly indexed with grip strength and forced expiratory volume [25,26]. In this study, we used continuous information on grip strength and FEV1 as indicators of physical fitness. *Grip strength* was measured with a dynamometer (Smedley, ranging from 0 to 100 kg). Participants started with the dominant followed by the non-dominant hand, and were asked to grasp with as much force as possible. Three measurements for each hand were requested, with the highest value of each hand being selected for later analysis. *Forced expiratory volume in one second (FEV1)* was used as an overall indicator of lung function. We only analyzed spirometry measurements (using EasyOne Spirometer; ndd Medical Technologies) with sufficient measurement quality, fully in line with standard procedures following the guidelines of the American Thoracic Society [27]. *Height* was measured to the nearest 0.1 cm by using an electronic weighting and measuring station (seca 764, seca, Hamburg, Germany). *Estimated glomerular filtration rate* (eGFR) was calculated according to the GFR-FAS formula (glomerular filtration rate computed by the full age spectrum equation). [28]. *Morbidity* was assessed as part of the medical examinations by physicians at the Charité university hospital Berlin. Diagnoses were obtained through participant reports, with select diagnosis (e.g., diabetes mellitus) being verified by additional (blood-laboratory) tests (for details, see [23]). Diagnoses were used to compute a morbidity index largely based on the categories of the Charlson index, which is a weighted sum of moderate to severe, mostly chronic physical illnesses, including cardiovascular (e.g., congestive heart failure), cancer (e.g., lymphoma). Each disease is assigned a number based on the severity of the condition (e.g., 1 for myocardial infarct, diabetes, ulcer or chronic liver disease, 2 for diabetes with end organ damage, tumor or lymphoma, 3 for moderate or severe liver disease, etc.; for details, see [29,30].

### Global cognitive functioning

The cognitive battery of BASE-II included the assessment of multiple tasks. Here, we focus on three main cognitive abilities: episodic memory (EM; indicated by Verbal Learning and Memory Test, Face–Profession Task, and Scene Encoding), working memory (WM; indicated by Letter Updating, Number-N-Back, and Spatial Updating), and fluid intelligence (Gf; indicated by Figural Analogies, Letter Series, and Practical Problems). The chosen tasks varied in procedures and content, consisting of items that relate to verbal, numerical, or figural–spatial information. The indicators consisted of the sum of correct responses (Letter Updating, Number-N-Back, Verbal Learning, Object Location, Practical Problems, Letter Series, Figure Analogies), averaged percentages of correct placements (Spatial Updating) and hits minus false alarms (Scene Encoding, Face Profession) with higher scores indicating better memory performance (for more information, refer [2] and [3]).

### Sociodemographic measures and time intervals

We also included several sociodemographic measures in our analysis. Age on the medical assessment was measured in years since birth, sex was coded as (1) for females and (2) for males. Education was measured in number of years. The objective health variables were collected about 1 year prior to cognitive testing (mean time difference in years = 1.2 years; SD = 0.80). To control for individual differences in time elapsing between measurements, we included the time interval as a control variable in the cognitive analyses. Time intervals between medical and cognitive assessments were calculated in months for each participant.

### Data preparation

To evaluate the latent factor structure of *metabolic load* and cognitive functioning, we conducted CFA using Mplus ([4]; Version 7). To account for missing data, we used full information maximum likelihood algorithm which is implemented in the Mplus software.

### Structural equation modeling approach

We used structural equation modeling (SEM) to investigate the relations between MetL, objective health, and cognitive performance for two reasons. First, SEM enables us to move beyond modeling at the manifest variable level to modeling the cognitive variables at the construct level, namely, on latent variables for MetL, WM, EM, and Gf. In doing so, we can account for and partial out the effects of measurement error, which enhances the validity of our analyses [31]. Second, SEM offers a generic framework to formalize and test our hypotheses about the potential interrelations of objective health, cognition and MetL. Our focal research question was whether MetL affects health and cognitive performance in healthy older adults. Due to the cross-sectional and observational nature of this data set, we cannot clarify the direction of effects. We aimed to address the question whether the relations between MetL, health and cognition is comparable across gender by means of a multi-group SEM analysis.

### Confirmatory factor analysis of cognition and metabolic load

We tested the statistical models in accordance with guidelines for proper execution of CFA techniques [32]. We relied on standard indices such as the root mean square error of approximation (RMSEA), standardized root mean square residual (SRMR), and the comparative fit index (CFI). Commonly accepted thresholds indicating good model fit are RMSEA≤0.05, SRMR≤0.05, and CFI≥0.95 [33,34].

Model of cognition: We applied a previously established and validated latent factor model of cognitive functioning within the whole sample which represents individual differences in WM, EM, and Gf [2].

Model of metabolic load: Based on previous empirical and conceptual work (e.g. Alberti et al. 2009), we hypothesized that one common factor would describe the relationship of cardio-metabolic components of *metabolic load* best, defined by the following indicators: waist circumference, triglycerides, fasting blood glucose (BG1), HDL, and systolic and diastolic blood pressure. All indicators and covariates were standardized to avoid problems in model estimation within the whole sample. Next, to determine whether the loadings of the single indicators differ and the *metabolic load* model is comparable between males and females we applied measurement invariance tests.

Measurement invariance of metabolic load: In order to determine whether the influences of all indicators originally selected might show sex-specific differences in the loadings on the *metabolic load* factor, we applied multi-group models to test measurement invariance of the *metabolic load* model. Measurement invariance tests evaluate the degree to which measurements conducted in different groups of people (here: male/female) reflect measurement of the same attributes [35,36]. The test of weak invariance is the least restrictive. Good fit indices indicate that groups do not differ in their factor structure and factor loadings. Testing of strong invariance requires all assumptions for weak and the same item intercepts across groups. Measurement equivalence of *metabolic load* was established by calculating the difference between the CFI-values of the two models (male & female) and accepting equivalence if DCFI<0.1 (as suggested by [36]).

### Predicting morbidity and physical health from metabolic load

Based on well-established associations between MetS with physical fitness and disease burden [3,4,8,14,37], we selected the following objective health parameters: lung function, grip strength, measures of morbidity, and GFR to validate sex-specific predictive value of the *metabolic load* factor. In order to investigate simultaneous associations between *metabolic load*, lung function, grip strength, morbidity, and GFR we set up a multi-group regression model for males and females, controlling for age, height, and years of education.

### Predicting cognitive functioning from metabolic load

We used latent regression analyses to investigate how EM, WM, and Gf relate to *metabolic load* as a function of sex. Missing data were handled using a full information maximum likelihood approach as implemented in the Mplus software. We set up a multi-group structural equation model to test whether associations between the three-factor model of cognition and *metabolic load* varied across sex. In addition, three covariates were entered to the SEM as exogeneous predictors of cognition and *metabolic load*: age, years of formal education, and the time difference between the medical and cognitive sessions.

### Predicting morbidity and physical health and cognitive functioning from single indicators of MetS

To determine whether *metabolic load* was a more comprehensive predictor of health and cognitive functioning we conducted multiple regression analysis within a multi-group structural equation model as described above but only by entering the observed scores of the four individual components into the regression models simultaneously. Covariates included in this model were age, years of formal education regressed on all indicators. Height was used to control for body size on grip strength and lung function.

Differences between MetS/no-MetS groups: Additionally, we applied independent samples-t-tests to look for differences in health and cognitive outcomes by using Alberti-cut-offs in our sample.

## Results

The sample characteristics and their differences regarding sex are summarized in Table 1. All demographic variables and indicators showed significant differences between male and female, except systolic and diastolic blood pressure. In summary, males showed higher age, more years of education and larger height (all *p*´s < .005). The groups also differed in all outcome variables, except eGFR and episodic memory (all *p*´s < .005, see Table 1).

**Table 1:** Descriptive statistics and sex-specific differences among study variables.

	*Men*		*Women*		*t-test*	
	*Mean*	*SD*	*Mean*	*SD*	*t*	*p*
(1) Age (60–84 years)	69	3.7	68.5	3.5	2.82	.005**
(2) Education (7–18 years)	14.5	2.8	13.7	2.8	5.03	.000**
(3) Height (cm)	174.9	6.2	162.6	6.0	38.83	.000**
(4) Date difference (weeks)	20	13.4	28	13.6	-11.4	.000**
(5) Waist circumference (cm)	99.6	7.8	102.1	35.1	-2.55	.000**
(6) Triglycerides (mg/dl)	121.1	73.8	104.7	50.9	5.12	.000**
(7) Fasting glucose (mg/dl)	100.1	23.6	92.2	15.0	7.62	.000**
(8) HDL-cholesterol (mg/dl)	55.0	14.5	69.3	4.3	-18.3	.000**
(9) Systolic blood pressure (mmHg)	149.7	60.2	153.4	89.6	-1.01	.314
(10) Diastolic blood pressure (mmHg)	88.7	62.3	92.1	94.3	-.486	.432
(11) MetL factor^	.04	.2	-.04	.1	10.26	.000**
*Morbidity & Physical Fitness*						
(12) Morbidity index	1.3	1.3	1.2	1.2	2.06	.039*
(13) FEV1 (ml)	2974.2	656.7	2130.7	493.9	23.01	.000**
(14) Grip Strength (kg)	37.6	6.1	23.3	4.3	51.6	.000**
(15) eGFR (ml/min/1.73 m^2^)	70.5	10.7	68.3	10.5	.203	.839
*Cognitive Functioning*						
(12) Working Memory^	0.5	5.8	-.05	5.2	-.897	.370
(13) Episodic Memory^	-0.1	1.2	.03	1.1	3.18	.002**
(14) Fluid Intelligence^	0.2	1.4	-.15	1.3	4.03	.000**
*MetS Diagnosis cut-off groups in BASE-II*	*Men*		*Women*			
	MetS	no-MetS	MetS	no-MetS		
	339 (43%)	449 (57%)	255 (31.1%)	566 (68.9%)		

*Note*. *N* = 1609 (men: *n* = 788; women: *n* = 821). FEV1 = forced expiratory volume in 1 second. eGFR = estimated glomerular filtration rate.

^factor score.

*p > .05.

**p > .001.

The intercorrelations of the variables under study are presented in Table 2. The indicators used to define *metabolic load* showed a moderate association among each other, suggesting that they represent distinct but correlated components of *metabolic load*. In our sample, 594 participants (31% female) were diagnosed with MetS according to Alberti et al. 2009 (see Table 1).

**Table 2:** Intercorrelations of variables under study.

	(1)	(2)	(3)	(4)	(5)	(6)	(7)	(8)	(9)	(10)	(11)	(12)	(13)	(14)	(15)	(16)	(17)	(18)
(01) Age (60–84 years)	--	-.055	-.125**	-.188**	-.054	-.062	-.033	-.044	-.025	-.069	-.030	-.166**	-.167**	-.080*	-.203**	-.203**	-.183**	-.034
(02) Education (7–18 years)	-.055	--	-.149**	-.058	-.083*	-.015	-.090*	-.028	-.003	.004	-.032	-.063	-.034	-.004	-.368**	-.345**	-.388**	-.084*
(03) Height (cm)	-.125*	-.149**	--	-.011	.214**	.059	-.026	-.016	-.066	-.048	-.006	-.258**	-.275**	-.051	-.160**	-.129**	-.143**	-.062
(04) Date difference	-.188**	-.058**	-.011	--	-.091*	.017	-.037	-.024	-.048	-.070	-.000	-.174**	-.077*	-.107**	-.087*	-.086*	-.097*	-.013
(05) Waist circumference (cm)	.054	-.083*	-.241**	-.091*	--	.224**	-.185**	-.270**	-.067	-.070	-.160**	-.038	-.065	-.016	-.070	-.033	-.082	-.403**
(06) Triglycerides (mg/dl)	-.062	-.015	-.059	-.017	-.224**	--	-.210**	-.414**	-.009	-.019	-.166**	-.033	-.022	-.078*	-.040	-.033	-.065	-.440**
(07) Fasting glucose (mg/dl)	-.033	-.090*	-.026	-.037	-.185**	.210**	--	-.094*	-.002	-.006	-.242**	-.106*	-.065	-.063	-.026	-.000	-.060	-.401*
(08) HDL-cholesterol (mg/dl)	-.044	-.028	-.016	-.024	-.270**	-.414**	-.94*	--	-.048	-.036	-.152**	-.047	-.039	-.021	-.079*	-.081*	-.086*	-.421**
(09) Systolic blood pressure (mmHg)	-.025	-.003	-.066	-.048	-.067	.009	-.002	-.048	--	-.967**	-.014	-.038	-.101**	-.006	-.005	-.011	-.009	-.043
(10) Diastolic blood pressure (mmHg)	-0.07	-.004	-.048	-.070	-.070	.019	-.006	-.036	-.967**	--	-.031	-.129**	-.090*	-.012	-.004	-.011	-.007	-.033
(11) Morbidity index	-.047	-.032	-.006	-.000	-.160**	.166**	-.242**	-.152**	-.014	-.031	--	-.154**	-.109**	-.053	-.040	-.036	-.037	-.247**
(12) FEV1 (ml)	-.17**	-.063	-.258**	-.174**	-.038	-.033	-.106**	-.047	-.038	-.129**	-.154**	--	-.242**	-.033	-.112*	.130**	-.129*	-.049
(13) Grip Strength (kg)	-.17**	-.034	-.275**	-.077*	-.065	.022	-.065	-.039	-.101**	-.090*	-.109**	-.242**	--	-.010	-.153**	-.146**	-.154**	-.020
(14) eGFR (ml/min/1.73 m^2^)	-.33**	-.035	-.083*	-.022	-.056	-.146**	-.008	-.097**	-.025	-.038	-.013	-.006	-.081*	--	-.056	-.041	-.052	-.029
(15) Working Memory^#^	-.20**	-.345**	-.160**	-.070	-.033	-.040	-.026	-.079*	-.005	-.004	-.040	-.112**	-.153**	.011	--	-.869**	-.952**	-.016
(16) Episodic Memory^#^	-.20**	-.368**	-.129**	-.033	-.070	-.033	-.000	-.081*	-.011	-.011	-.036	-.130**	-.146**	-.025	-.869**	--	-.783**	-.014
(17) Fluid Intelligence^#^	-.18**	-.388**	-.143**	-.083	-.082	-.065	-.060	-.086*	-.009	-.007	-.037	-.154**	-.154**	-.007	-.952**	-.768**	--	-.053
(18) MetL factor^#^	-.067	-.033	-.050	-.026	-.286**	-.963**	-.371**	-.579**	-.002	-.008	-.209**	-.019	-.008	-.145**	-.056	-.047	-.084*	--

*Note*. *N* = 1609 (men: *n* = 788; women: *n* = 821). FEV1 = forced expiratory volume in 1 second. eGFR = estimated glomerular filtration rate;

^#^MetL and cognitive functioning indicators are represented as latent variables. Men are represented below and women above the diagonal.

*p > .05.

**p > .001

### CFA of metabolic load

The CFA of Model 1 resulted in a good Model fit (χ^2^(8) = 9.7; CFI = 1.0; RMSEA = 0.012; SRMR = 0.010). Waist circumference and triglycerides as well as systolic and diastolic blood pressure were allowed to be correlated. The selected indicators loaded significantly on the *metabolic load* factor (standardized estimates of path loadings: waist = .194; HDL = -.551, TG = .789; BZP1 = .366; p< .05) except systolic and diastolic blood pressure (standardized estimates of path loadings for RRsys: .038; RRdiast = .039 p >.200), which is not unusual and in accordance with prior findings [38,39]. Following guidelines for confirmatory factor analyses, we thus excluded blood pressure as indicators of *metabolic load*.

**Fig 1:**
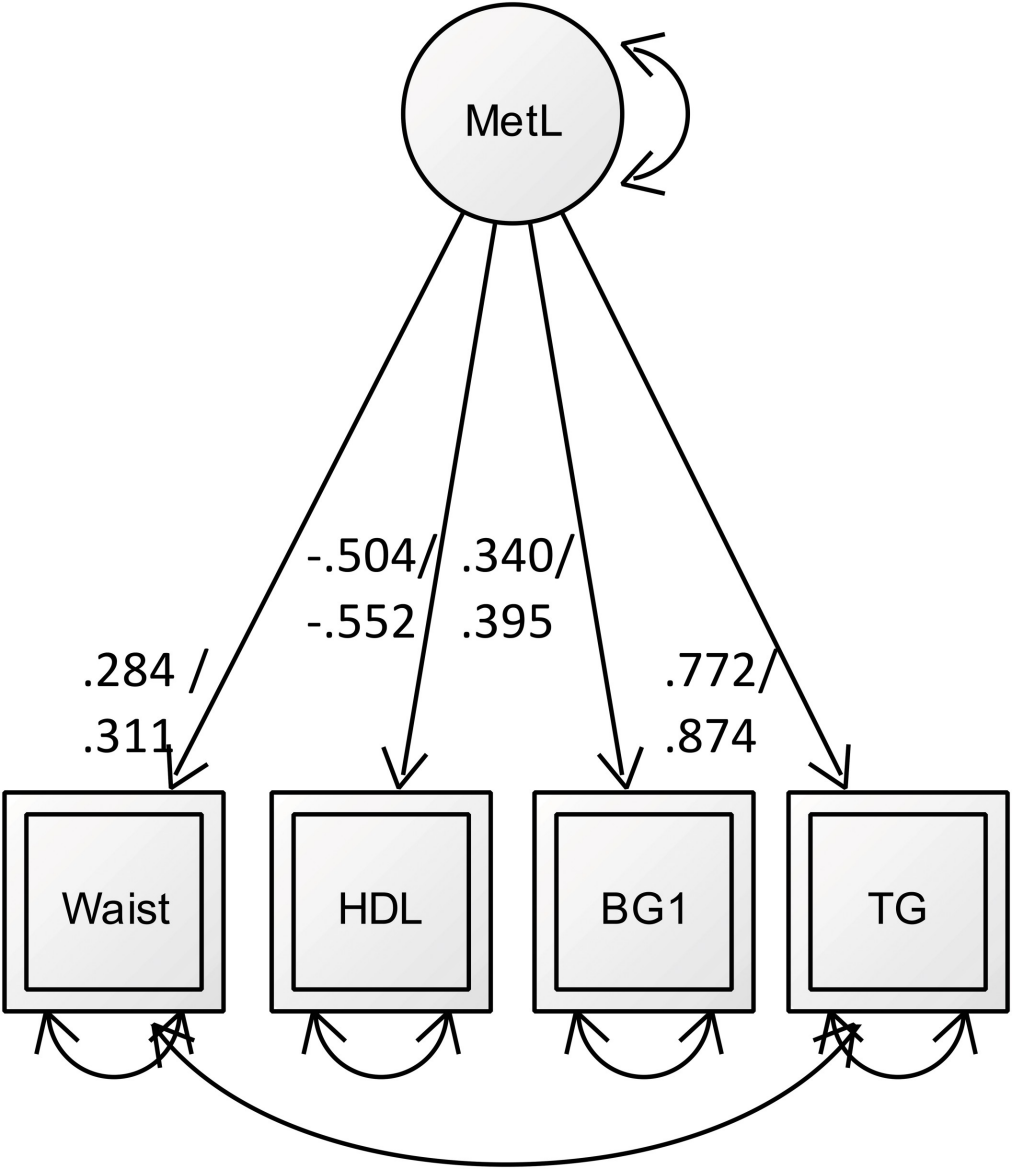
A simplified illustration of CFA results. One-factor solution of metabolic load. The significant standardized factor loading’s (all p’s < .05) are depicted in numbers on single-headed arrows. Numbers on arrows refer to the factor loading’s for the male (first) and female (second) group.

In the final Model 2 all remaining indicators loaded significantly on the *metabolic load* factor and provided a good model fit (χ^2^(2) = .23; CFI = 1.00; RMSEA = 0.00; SRMR = 0.004; see Fig 1; factor loadings are separated for the male and female group). Error terms between waist circumference and triglycerides were allowed to be correlated.

Measurement equivalence of metabolic load was established by calculating the difference between the CFI-values of the two models (male & female) and accepting equivalence if DCFI<0.1 (as suggested by Cheung and Rensvold, 2002).

### Predicting morbidity and physical function with metabolic load

**Fig 2:**
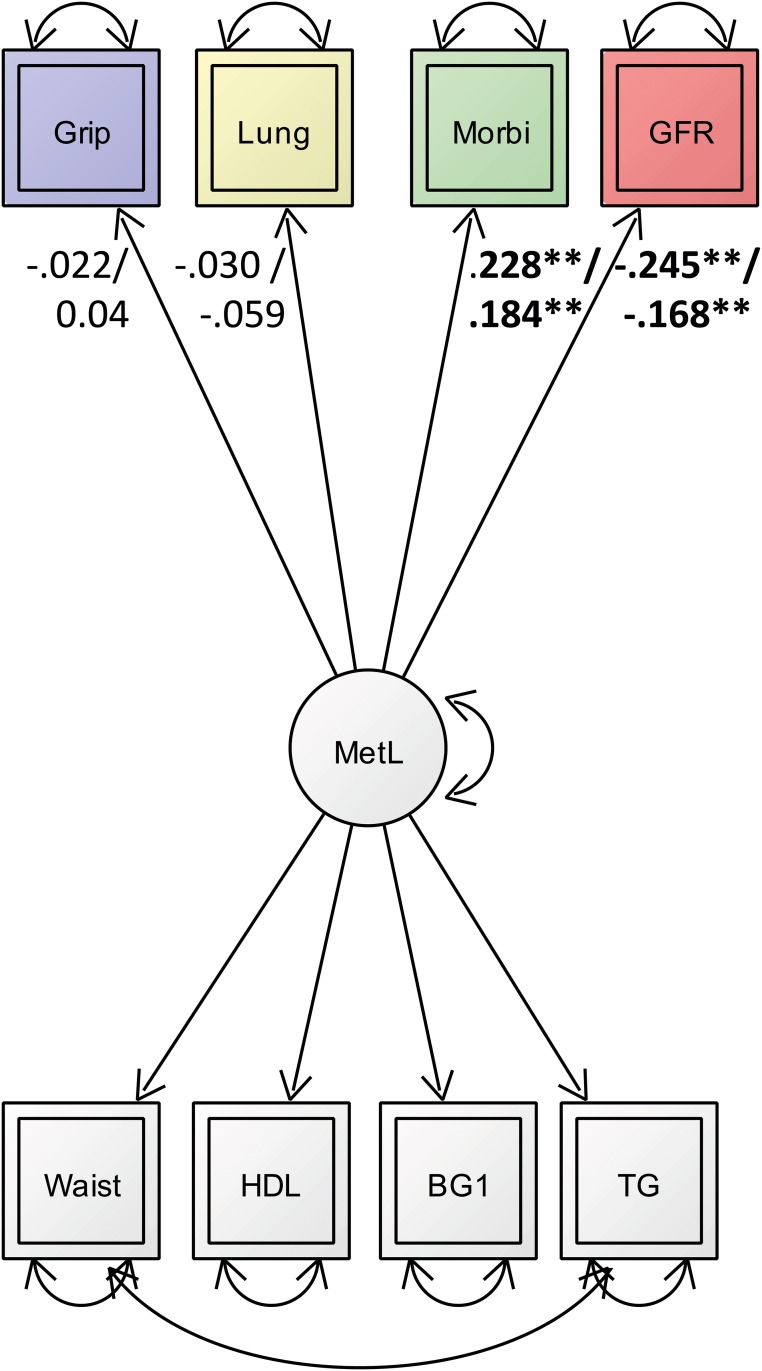
Latent regression model between the metabolic load factor and indicators of physical health (grip strength, lung volume ratio, morbidity, and GFR). Double headed arrows presenting covariance and error terms between and of indicators respectively. Significant standardized regression estimates are depicted in bold numbers on single-headed arrows. Numbers on arrows refer to the male (above) and female (below) group. *p > .05; **p > .001.

As depicted in Fig 2 and Table 2, *metabolic load* was significantly associated with morbidity, both in males and females (***ß***_***male***_ = 0.228, and ***ß***_***female***_ = 0.184, *p* = 0.00; model fit: χ^2^(60) = 183; CFI = 0.890; RMSEA = 0.53; SRMR = 0.048). From the physical fitness indicators only GFR showed a negative significant association, both in males and females (***ß***_***male***_ = -0.245, and ***ß***_***female***_ = -.168, *p* = 0.00). Grip strength and lung function (FEV1) were not associated with *metabolic load* neither in the group of males nor females (*p* > 0.05).

### Predicting cognitive function with metabolic load

**Fig 3:**
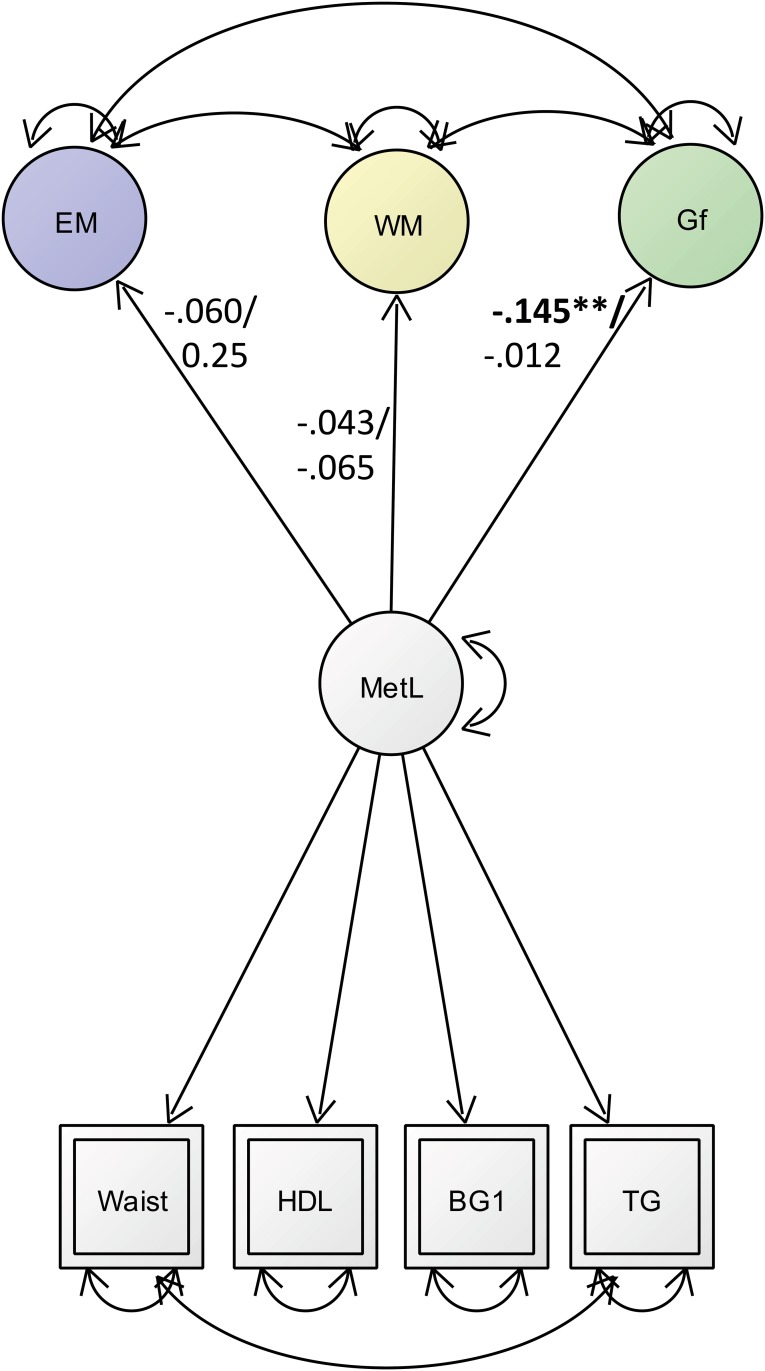
Latent regression model between the three cognitive factors (EM = episodic memory factor; WM = working memory factor; GF = Fluid Intelligence) and the metabolic load factor. The covariance between latent factors are depicted on double headed arrows; Significant standardized regression estimates are depicted in bold numbers on single-headed arrows. Numbers on arrows refer to the male (first) and female (second) group. *p > .05; **p > .001.

We found a negative association between *metabolic load* and *fluid intelligence* (Gf) only in the male group (***ß*** = -0.145; *p* = 0.019; model fit: χ^2^(204) = 387; CFI = 0.949; RMSEA = 0.38; SRMR = 0.037), while the other cognitive domains tested, *episodic memory* (EM) and *working memory* (WM) were not associated with *metabolic load* (Fig 3).

### Associating health and cognitive outcomes with single MetS indicators

The predictions of the five single indicators on (i) health and (ii) cognition are summarized in the upper part of Table 3. In men, fasting glucose was negatively associated with FEV1 (-.114; p < .00) and triglycerides (-.179; p < .00) and positively with morbidity (.240; p < .00) after controlling for the effects of age, years of education, and height. In women, waist circumference was negatively correlated with FEV1 (-.279; p < .00) and GFR (-.263; p < .00) and fasting glucose was positively correlated with morbidity index (.206; p < .00) and GFR (.166; p < .00). For cognition, fasting glucose was negatively correlated with EM in women, after controlling for the effects of age, years of education, and testing date difference. Table 4 depicts differences between MetS diagnosed and healthy groups. By applying the conventional cut-off criteria in our sample, the groups only differed in age, BMI and morbidity (all *p’s* < .05).

**Table 3:** SEM results: Standardized regression coefficents (ß) predicting physical and cognitive functioning from individual and latent MetL components separately for male and female participants.

SEM	*Men*							*Women*						
	Grip	FEV1	Morbi	GFR	WM	EM	Gf	Grip	FEV1	Morbi	GFR	WM	EM	Gf
*Single Indicators*														
(1) Waist circumference (cm)	-.043	.011	-.028	-.034	-.057	-.032	-.057	-.023	-.279**	.066	-.089	-.018	-.036	-.048
(2) HDL-cholesterol (mg/dl)	-.023	.037	-.070	-.094	-.005	-.045	-.075	-.028	-.000	-.067	-.074	-.075	-.053	-.066
(3) Fasting glucose (mg/dl)	-.069	-.114**	.240**	-.008	-.019	-.054	-.051	-.001	-.022	.206**	-.166**	-.011	-.156**	-.001
(4) Triglycerides (mg/dl)	-.024	.009	-.073	-.179**	-.034	-.057	-.032	-.022	-.003	.016	-.263**	-.025	-.050	-.095
*Covariates*														
Age (years)	-.132**	-.105*	-.026	-.350**	-.173**	-.205**	-.130**	-.102**	-.230**	-.095	-.356**	-.121**	-.170**	-.146**
Years of education	-.058	-.004	-.001	-.074	-.343**	-.360**	-.409**	-.058	-.033	-.005	-.015	-.268**	-.057	-.332**
Height	-.246**	-.238**	--	--	--	--	--	-.278**	.343**	--	--	--	--	--
Testing date difference	--	--	--	--	-.058	-.041	-.070	--	--	--	--	-.017	-.029	-.004
Metabolic load*	-.013	-.057	.228**	-.245**	-.043	-.060	-.145**	.033	-.047	.184**	-.168**	-.065	-.025	-.012
*Covariates*														
Age	-.124**	-.173**	-.001	-.343**	-.173**	-.202**	-.119**	-.103**	-.094	-.101**	-.363**	-.119**	-.182**	-.150**
Years of education	-.057	-.023	-.016	-.071	-.346**	-.356**	-.418**	-.059	-.000	-.005	-.012	-.269**	-.265**	-.333**
Height	.256**	-.117**	--	--	--	--	--	-.279**	-.123**	--	--	--	--	--
Testing date difference	--	-.--	--	---	-.065	-.040	-.081	--	---	--	--	-.018	-.049	-.005

*Note*. FEV1 = forced expiratory volume in 1 second.

*p > .05.

**p > .001

**Table 4:** Independent 2 sample t-test of group differences of variables within the male group under study separated for MetS / no-MetS.

	MetS-group male/female	no-MetS group male/female	*ß*	*p*
(1) Age	56.8 /18.7	66.1/10.9	167.4	.000**
	58.1 /17.7	66.3/10.1		
(2) Education	14.7 /2.7	14.3/2.9	222.2	.683
	14.2 /2.8	13.7/2.7		
(3) BMI	25.1 /3.2	28.8/3.8	223.9	.047*
	24.4 /4.01	29.0/4.5		
(4) Morbidity Index	24.80 /1.1	21.4/1.4	116.1	.001**
	24.81/1.1	21.2/1.4		
(5) FEV1 (ml)	24.731/.1	22.8/.1	222.9	.338
	24.742/.1	22.8/.1		
(6) Grip Strength (kg)	38.9/7.1	38.4/6.0	222.04	.954
	24.8/4.9	23.5/4.8		
(7) eGFR (ml/min/1.73 m^2^)	71.8/10.7	69.4/10.2	222.92	.329
	68.8/10.7	69.4/10.2		
(8) Working Memory^#^	24.4/5.9	22.9/5.5	221.2	.270
	2 -.2/5.1	2 -.6/5.5		
(9) Episodic Memory^#^	2 -.1/1.2	22.1/1.2	221.3	.248
	24.07/1.0	22.02/1.8		
(10) Fluid Intelligence^#^	24.2/1.3	22.2/1.4	221.9	.159
	2 -.12/1.2	2 -.19/1.4		

^#^Metabolic load and cognitive functioning indicators are represented as factor scores; eGFR = estimated glomerular filtration rate; Numbers in bold indicate significant differences.

*p > .05.

**p > .001

## Discussion

We hypothesized that the established one-latent factor model of *metabolic load* more comprehensively reflects the severity of metabolic condition in our sample of mostly healthy older adults, in contrast of single indicators of MetS according to Alberti et al [1] and consequently might be a more fine-grained approach to use in more healthy larger samples regarding established associations with physical fitness, morbidities and cognitive function. In order to investigate the hypothesis, we carried out cross-sectional analysis of 1,609 older BASE-II participants. We first selected indicators based on the joint statement on MetS of the International Diabetes Federation Task Force [1], namely waist circumference, triglycerides, fasting blood glucose, high-density lipoprotein cholesterol and systolic /diastolic blood pressure to define in a CFA a latent factor for *metabolic load*. The use of CFA is particularly suitable to reflect the interplay between these mechanisms, and is rather independent of single MetS constituents that may be predominant in some subjects diagnosed with MetS according to cut-off values. We successfully validated the psychometric structure of a one-latent metabolic load factor by the best fitting factorial solution, suggesting that in a relatively healthy older adult sample the indicators we selected significantly contribute to the latent *metabolic load* factor model. Interestingly, triglyceride and high density lipoproteins showed high loadings on *metabolic load* suggesting that blood lipids may drive the associations between, metabolic load, health and cognition in our sample.

Next, we tested measurement equivalence of the metabolic load factor and found that the indicators chosen to define *metabolic load* do not differ across sex by showing comparable loadings. By using the CFA approach, we were able to demonstrate that the use of continuous information of MetS indicators (most commonly used as cut-off criteria) is a more comprehensive and valid approach to provide a continuous metabolic severity measure for each participant without using cut-offs.

This finding reflects the common idea of MetS that the clustering of single components show common pathomechanisms behind the development of the MetS, which seems also be true in healthy older adults.

The concept of the MetS is based on a complex interplay between metabolic, inflammatory and endocrine alterations, which result in a common occurrence of abdominal obesity, insulin resistance, dyslipidemia and hypertension.[1,17] Particularly insulin resistance is considered as a pathogenetically significant basis.[17] One consequence of insulin resistance is dyslipidemia, which is also manifest in type 2 diabetes. Hypertriglyceridemia is one of the early and frequently detectable consequences of insulin resistance.[40] IR promotes the increase of free fatty acids (FFA), since insulin is involved in the inhibition of lipolysis in the liver with a subsequent increase in triglyceride levels.[41] Triglyceride-HDL particles can be easily degraded by the hepatic lipase in the liver, resulting in lower HDL-C levels. The same effect on the lipid profile is observed in abdominal obesity, as hypertrophy of adipocytes is also associated with elevated levels of FFA [17]. Obesity is mainly driven by lifestyle habits (dietary habits and physical inactivity), however, there are also age-related (endocrine) changes and genetic aspects, which lead to increased fat storage. Recurring activation of insulin secretion due to dietary habits promotes insulin resistance.[42] Moreover abdominal obesity favors insulin resistance as a result of the effect of acute elevated FFA on skeletal muscle and glucose uptake in muscle.[43] Thus, it becomes obvious that pathomechanisms involved in the development of IR, dyslipidemia and obesity are closely intertwined and mutually reinforce and influence each other. Nevertheless, obesity does not inevitably lead to IR, dyslipidemia or hypertension, and these phenotypes can also occur in normal weight people. In addition, the development of metabolic impairment differs according to sex [17–19].

Even though the link between the above mechanisms to hypertension is not so apparent, there is a heaped occurrence of hypertension when dyslipidemia, IR and abdominal obesity occur. One explanation is an activation of the sympathetic nervous system and consequent vasoconstriction driven by IR. In addition, a renin-angiotensin system has been proposed in adipocytes, providing a link to hypertension [44]. As abdominal obesity and IR closely interact and may act on the development of hypertension, this is also the basis for the connection to dyslipidemia with hypertension. Nevertheless, systolic and diastolic blood pressure did not load on the MetL factor in the current analysis. While this is contradictory to several studies which have shown that blood pressure is part of a one factor structure together with the anthropometric and metabolic variables constituting the MetS [21,38,39,45–47]. However, multiple studies reported that they found at least two latent factors underlying the MetS, with blood pressure representing a separate factor (e.g. [39]). Essential hypertension is the most common form of hypertension in the general population, particularly in the old. A possible explanation why hypertension did not load on the MetL factor in BASE-II might be, that hypertension in our comparably old cohort is based on other mechanisms than the one discussed above. Two or more factor solutions in the context of MetS have been interpreted with respect to different pathomechanisms before [38]. Moreover, the development of hypertension seems to be a late consequence of the mechanisms involved in MetS and a loading of blood pressure was mainly described in younger subjects than the ones studied here. Additionally, activation of the sympathetic nervous system and renin-angiotensin system in the old [48] and its response to stimuli is changing, so these mechanisms might have less effect in the old.[49]

In a second set of analyses, we aimed to validate the *metabolic load* factor in regard to its predictive validity of health and cognition. We applied structural equation modeling and found sex-specific associations between *metabolic load* and physical fitness, morbidities and cognition. Metabolic Load was associated with lung function and morbidity in both, males and females.

In previous studies, different definitions of physical fitness have been used to assess its relationship with metabolic impairment and MetS, respectively. We decided to use FEV1 and grip strength to reflect physical fitness, as reported previously [25]. Overall, the association between MetS and reduced physical fitness is consistent through the studies, showing lower FEV1s in subjects with metabolic impairment and MetS, independent of definitions used [50,51]. This has also been confirmed in participants of BASE-II [8]. However, we have shown that this association is partly driven by single MetS components such as obesity and insulin resistance [8,51]. Likewise, the relationship between MetS and the prognostically important low grip strength has been seen before [5,10] but again, might be driven more by certain MetS phenotypes such as insulin resistance or obesity [37]. Otherwise, findings might be driven by single parameters of MetS that dominate the associations or are predominant in the study population. With regard to our study subjects, we previously reported that insulin resistance and abdominal obesity were predominant in our subjects with MetS, which might vary in other populations and lead to different MetS-phenotypes [16,37].

More interestingly, our results showed different associations between males and females in cognitive domains. In males we found specific associations between *metabolic load* and fluid intelligence whereas in the group of females *metabolic load* was associated with episodic memory performance. In contrast to the established associations between metabolic risk factors and health, results regarding the relationship between metabolic impairment or MetS and cognitive function are more complex. The majority of studies found negative associations between MetS and cognition or brain structure, but there is again evidence that single MetS parameters might be responsible for this association [52,53,54]. Tournoy et al. reported no association between MetS and cognitive domains, but an association of diabetes and cognitive impairment [53]. Other researchers found associations between impaired cognitive function and increased blood pressure but not with MetS, while other studies revealed no associations or only associations with the number of MetS components above the cut-off [55–57]. Applying the metabolic score factor, we found negative associations of *metabolic load* and Gf in men, which is in line with the majority of previous findings [14,58]. This indicates that the *metabolic load* factor defined here may be suitable to detect early cognitive alterations possibly associated with subtle metabolic changes in a healthy elderly population. This parameter, which includes the indicators used within the concept of MetS may be applied in clinical research in order to identify disease developments in early stages of metabolic impairment and MetS, respectively. The continuous information of the single metabolic indicators merged on *metabolic load* factor is independent of cut-off values and may reflect the development of metabolic impairment based on the theoretical concept of MetS.

## Conclusion

We established a *metabolic load* factor as a precursor of metabolic impairment in a sample of older adults originating from the BASE-II study to investigate associations with health and cognition usually found in MetS. By using a one-latent-factor model of metabolic load we were able to replicate these known associations. Moreover, we found sex-specific associations which were more pronounced in males. We were able to demonstrate associations between morbidity, physical health and cognition and *metabolic load*, independent of fixed cut-off values. This approach seemed to be sufficient to qualify early metabolic impairment in rather healthy older adults and might help longitudinally to understand the evolution of MetS. The findings underline the importance of early lifestyle and pharmacological interventions that might set up differently for males and females. This approach might help to improve the understanding of the association between early stages of MetS, health and cognition in different study populations.
